# Determination of Odor Release in Hydrocolloid Model Systems Containing Original or Carboxylated Cellulose at Different pH Values Using Static Headspace Gas Chromatographic (SHS-GC) Analysis

**DOI:** 10.3390/s130302818

**Published:** 2013-02-27

**Authors:** Sang Mi Lee, Gil-Ok Shin, Kyung Min Park, Pahn-Shick Chang, Young-Suk Kim

**Affiliations:** 1 Department of Food Science and Engineering, Ewha Womans University, Seoul 120-750, Korea; E-Mails: arnica78@hanmail.net (S.M.L.); sgo6280@hanmail.net (G.-O.S.); 2 Department of Agricultural Biotechnology, Seoul National University, Seoul 151-742, Korea; E-Mails: sgrin1979@gmail.com (K.M.P.); pschang@snu.ac.kr (P.-S.C.)

**Keywords:** cellulose, regio-selectively carboxylation, release, hydrocolloid, static headspace gas chromatographic (SHS-GC) analysis

## Abstract

Static headspace gas chromatographic (SHS-GC) analysis was performed to determine the release of 13 odorants in hydrocolloid model systems containing original or regio-selectively carboxylated cellulose at different pH values. The release of most odor compounds was decreased in the hydrocolloid solutions compared to control, with the amounts of 2-propanol, 3-methyl-1-butanol, and 2,3-butanedione released into the headspace being less than those of any other odor compound in the hydrocolloid model systems. However, there was no considerable difference between original cellulose-containing and carboxylated-cellulose containing systems in the release of most compounds, except for relatively long-chain esters such as ethyl caprylate and ethyl nonanoate. The release from the original and carboxylated cellulose solutions controlled to pH 10 was significantly higher than that from solutions adjusted to pH 4 and 7 in the case of some esters (ethyl acetate, methyl propionate, ethyl propionate, ethyl butyrate, butyl propionate, ethyl caproate) and alcohols (2-propanol, 3-methyl-1-butanol), in particular, ethyl butyrate and 3-methyl-1-butanol. In contrast, the release of 2,3-butanedione from both the original and carboxylated cellulose solutions was increased at pH 4 and 7 compared to that at pH 10 by about 70% and 130%, respectively. Our study demonstrated that the release of some odorants could be changed significantly by addition of both original and carboxylated cellulose in hydrocolloid model systems, but only minor effect was observed in pH of the solution.

## 1. Introduction

Polysaccharides are widely used as thickening and stabilizing agents to modify the texture and appearance of foods, and they also replace fat in calorie-reduced foods [1]. Polysaccharides can also modify the rate and intensity of the release of odors in food through specific or non-specific binding of the odor molecules and the physical entrapment of odor molecules within the food matrix [2]. Accordingly, the addition of polysaccharides generally reduces odor release due to an increase in viscosity and/or by molecular interactions with the odor compounds in hydrocolloid model systems. The effects of polysaccharides on odor release in aqueous food systems have been extensively reviewed and have been applied to polysaccharides in hydrocolloid systems [3–5]. The physicochemical interactions that occur between the odor compounds and the other constituents of the food matrix, especially polysaccharides, play an important role in the retention or release of odor substances during the processing, storage, and consumption of foods [4].

Different analytical approaches—both static methods (e.g., headspace analysis) and dynamic methods (e.g., exponential dilution)—for determining odor volatility in the presence of different food components have been applied to gain insight into the interactions between odor compounds and the food matrix [6–8]. Static headspace analysis permits a measurement by gas chromatography of the odorous compound concentration in gas phase when the gas/liquid equilibrium is reached [9]. This method directly measures the volatility of odorants by measuring only their concentrations in the gas phases [10]. Static headspace analysis has been widely used to study the volatility of odorants in the presence of non-odor components. It has been employed to establish the partition coefficients of benzene, toluene, chlorobenzene and 2-butanone in a gas/liquid system without measuring the concentrations of odor compounds in both phases [11,12]. This technique has also been successfully applied to measurements of Henry's constant of odor compounds, and has been validated by comparing the obtained data with those found in the literatures [13,14]. Compared to static headspace methods, dynamic methods are additionally able to provide information on the temporal release of compounds. Temporal release is determined by both thermodynamic and kinetic data. In dynamic methods, the concentrations of the odor compounds in the gas phase decrease exponentially as a function of time which enables fast determination of the partition coefficients of odorants [15].

Cellulose, the most abundant natural β-(1→4)-D-polysaccharide, has attracted a wide range of interest in the food industry due to its biological activity, biocompatibility, structure-forming capacity, and benign environmental effects [16]. It has diverse applications as an anti-caking agent, stabilizer, dispersing agent, thickener, and gelling agent, but these are generally secondary to its most important use as a water-holding agent [17]. This macromolecule has three kinds of hydroxyl groups with different acidities/reactivities: Secondary OH at the C-2 position, secondary OH at the C-3 position, and primary OH at the C-6 position; these can form strong and various intermolecular and intramolecular hydrogen bonds [16]. However, cellulose is a water-insoluble fiber consisting of 2,000—14,000 residues. To overcome this drawback, diverse methods have been used to increase the solubility of polysaccharides. Ohno *et al.*[18] established an efficient method to enhance the solubility of β-glucan by sodium hypochlorite (NaClO) oxidation. Also, Cross *et al.*[19] reported on the biological activity of β-glucan oxidized by TEMPO (2,2,6,6-tetramethylpiperidin-1-yl)oxyl radical). However, this non-specific and random carboxylation of polysaccharides has led to the loss of their original function and structure. Chang and co-workers attempted a different approach to improve the water-solubility of polysaccharides by introducing specific carboxylation, thereby conserving the original linkage of the polysaccharides. They demonstrated that mild and selective oxidation of the polysaccharides resulted in a great improvement in the water solubility of the oxidized products, in addition to a preservation of their functionality and biological activity [20].

Although various applications of modified polysaccharides are expected in hydrocolloid systems, the effect of the selectively carboxylated polysaccharides on the release of odor compounds in these systems has never been studied. Therefore, the objective of our study was to examine how the addition of cellulose modified by regioselective carboxylation affects the release of 13 different odor compounds (ethyl acetate, methyl propionate, ethyl propionate, ethyl butyrate, butyl propionate, ethyl caproate, ethyl caprylate, ethyl nonanoate, 2-propanol, 3-methyl-1-butanol, 2,3-butanedione, *trans*-2-hexenal, and *cis*-4-decenal) from hydrocolloid model systems using static headspace gas chromatographic (SHS-GC) analysis. Also, the release of odor compounds according to pH values such as 4, 7, and 10 was investigated and compared in both hydrocolloid model systems. Our study could support the use and application of intact cellulose and carboxylated cellulose in food hydrocolloid systems.

## 2. Experimental Section

### 2.1. Materials

All chemical standard compounds (ethyl acetate, methyl propionate, ethyl propionate, ethyl butyrate, butyl propionate, ethyl caproate, ethyl caprylate, ethyl nonanoate, 2-propanol, 3-methyl-1-butanol, 2,3-butanedione, *trans*-2-hexenal, *cis*-4-decenal, and ethyl alcohol) used in this experiment were purchased from Aldrich (Milwaukee, WI, USA). Water was distilled and purified using a Millipore Q system (Millipore, Billerica, MA, USA) before use. The physicochemical properties of these odor compounds are listed in Table 1.

The 13 odor compounds were chosen on the base of their functional groups and log *P* values. In particular, aldehydes, alcohols and ketones, which could have hydrogen bonds with cellulose molecules, were included. Also, a series of esters were studied to investigate the effect of hydrophobicity on the release of odorants.

### 2.2. Procedure to Modify Cellulose by Regio-Selective Carboxylation

The regioselectively carboxylated cellulose was obtained by the following process: cellulose (3.566 g, 10 mmol) was dispersed in water (100 mL) and stirred. Oxidation was initiated by the addition of 2,2,6,6-tetramethylpiperidin-1-yl)oxyl radical (TEMPO, 0.1 mmol, 16 mg), sodium bromide (0.5 g), and 1.389 mol/L sodium hypochlorite solution (31.5 mL). The reaction was conducted at 25.0 ± 0.5 °C and pH 10.8. The pH was monitored and controlled with 0.5 mol/L sodium hydroxide using a pH-stat (Metrohm Ltd., Hensau, Switzerland). After one mmol hydroxide per mmol of primary alcohol was added, oxidation of cellulose was quenched by the addition of *n*-propanol (2.5 mL per 100 mL of solution), followed by neutralization with 4 mol/L hydrochloric acid. The oxidized product was precipitated by the addition of two volumes of *n*-propanol. The precipitate was washed using 5 to 6 volumes of *n*-propanol and dried *in vacuo* at 45 °C to produce a dry power. The primary hydroxyl group of cellulose was oxidized to a carboxylic group by reduction catalysts such as TEMPO, NaOCl, and NaBr [20].

### 2.3. Confirmation of the Carboxylation of Cellulose by ^13^C-NMR and IR Analyses

Proton-coupled ^13^C-NMR spectra were obtained to determine the structure of the oxidation product of cellulose using a Bruker AMX-500 NMR instrument (Bruker Co., Bremen, Germany). An oxidized sample (2.5 mg/mL) was dissolved using D_2_O in a capillary tube, and deuterated dimethyl sulfoxide was used as an internal standard. IR analysis was also performed on cellulose and oxidized cellulose. Dry powder was applied with potassium bromide pellet as a control. Spectra were collected using a FTS-135 (Bio-Rad Co., Cambridge, MA, USA) to elucidate the change of cellulose structure and confirm the formation of carboxyl group in oxidized cellulose.

### 2.4. Sample Preparation for Static Headspace Gas Chromatographic (SHS-GC) Analysis

One% solutions of original and carboxylated cellulose were prepared by dissolving in water at room temperature. Then, pH of each solution was adjusted to 4, 7, or 10, and then homogenized using a Vortex (Scientific Industries, INC. Bohemia, NY, USA) at room temperature for 30 sec.

The 13 odorants (ethyl acetate, methyl propionate, ethyl propionate, ethyl butyrate, butyl propionate, ethyl caproate, ethyl caprylate, ethyl nonanoate, 2-propanol, 3-methyl-1-butanol, 2,3-butanedione, *trans*-2-hexenal, and *cis*-4-decenal) were dissolved in ethyl alcohol at the concentration of 1.7% (w/w). This stock solution was added dropwise to water to obtain a final concentration of 420 mg/L for ester compounds and 1,680 mg/L for other odorants, and then diluted by water before use. Accordingly, the final concentrations of each odorant and cellulose in hydrocolloid solutions containing 13 odorants were 84 mg/L for ester compounds, 336 mg/L for other odorants, and 0.08% for cellulose. Then, 5 mL of each hydrocolloid solution was transferred into a headspace vials (22 mL). An aqueous solution without cellulose was also prepared as a reference solution (blank solution). The three different types of solutions (original cellulose, carboxylated cellulose, blank solution) containing all 13 odor compounds were used for static headspace gas chromatographic analysis. The final concentrations of each odorant added were determined considering their solubilities and stabilities in the hydrocolloid systems as well as the analytical reproducibilities and precisions.

### 2.5. Measurement of Odor Release from Hydrocolloid Model Systems by Static Headspace Gas Chromatographic (SHS-GC) Analysis

SHS-GC analysis was performed using a Varian CP 3800 gas chromatograph (Varian, Walnut Creek, CA, USA) equipped with a static headspace autosampler (Tekmar Dormann Headspace 7000, Cincinnati, OH, USA). The time to reach an equilibrium between the liquid and gas phases in the vial was determined to be 45 min at 37 °C. Samples were additionally kept at 37 °C for 15 min and agitated at 750 rpm for 1 min in the automated headspace unit connected to GC before injected. After the vials had been pressurized with carrier gas for 30 sec, 1 mL of the headspace sample was injected into a DB-5 capillary column (length: 30 m, internal diameter: 0.25 mm, film thickness: 0.25 μm; J&W Scientific, Folsom, CA, USA) for 1 min. Before sample was pressurized, sample was mixed for 1 min at mixing power 5 (mixing power range of headspace autosampler; (1–10)). Pressurizing and loop filling times were 0.5 min and 0.4 min, respectively. Both sample loop and line temperatures were 150 °C. The GC injector and detector temperatures were 230 °C and 290 °C, respectively, and helium (0.9 mL/min) was carrier gas. The GC oven temperature was maintained at 25 °C for 5 min and then programmed as follows: from 25 °C to 45 °C (5 min) at a rate of 1 °C/ min; from 45 °C to 110 °C (5 min) at a rate of 10 °C/min; from 110 °C to 230 °C (2 min) at a rate of 40 °C/min. For the flame ionization detector, air and hydrogen flow rates were 400 mL/min and 35 mL/min, respectively. The release of each odorant from hydrocolloid solution was measured by the peak area of each odor compound on GC chromatogram. The identification of 13 odorants was achieved by comparison of retention times of each odorant with those of authentic standard compounds on DB-5 column. This experiment was performed in triplicate.

### 2.6. Statistical Analysis

For all data of triplicate measurements, analysis of variance (ANOVA) was performed using the general linear model procedure to determine the significant differences between the samples. Duncan's multiple range test was carried out on the level of significance set at p < 0.05. ANOVA and Duncan's multiple range test were performed with SPSS (version11.0, Chicago, IL, USA).

## 3. Results and Discussion

### 3.1. Release of Odorants in Hydrocolloid Model Systems

**Figure 1.**
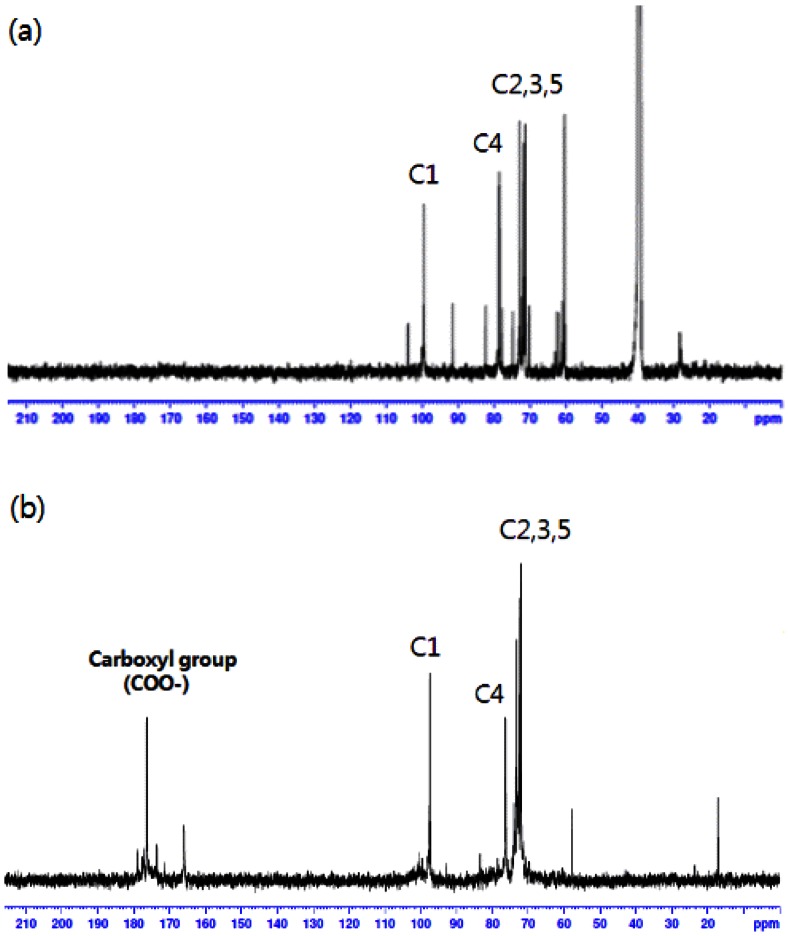
^13^C-NMR spectra of original cellulose (**a**) and carboxylated cellulose (**b**).

**Figure 2.**
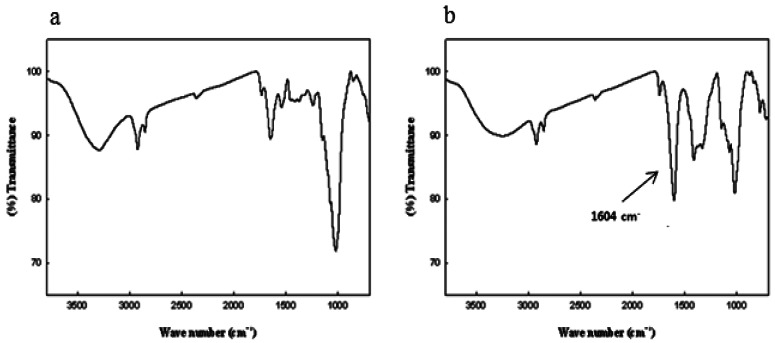
IR spectra of original cellulose (**a**) and carboxylated cellulose (**b**).

The carboxylation of cellulose was confirmed using proton coupled ^13^C-NMR and IR analyses (Figures 1 and 2). As one can see in Figure 1, the spectra of carboxylated cellulose showed new resonance at 180 ppm, which resulted from the formation of a carboxyl group. Also, no resonance was observed in the region of around 200 ppm, indicating that a ketone group was not formed during the oxidation reaction. Also, IR spectra of the cellulose and carboxylated cellulose are shown in Figure 2. IR spectra of carboxylated cellulose indicated a sharp peak at 1,604 cm^−1^, implying the presence of a C=O bond. The broad peaks in the range of 3,200–3,600 cm^−1^ were attributed to the presence of OH groups. This meant the secondary alcohol groups (–CHOH–) and ring structure of β–D-glucose molecules were intact and unchanged. If the secondary alcohol groups in cellulose were oxidized during the reaction, the peak in the 3,200–3,600 cm^−1^ range would not be observed.

**Figure 3.**
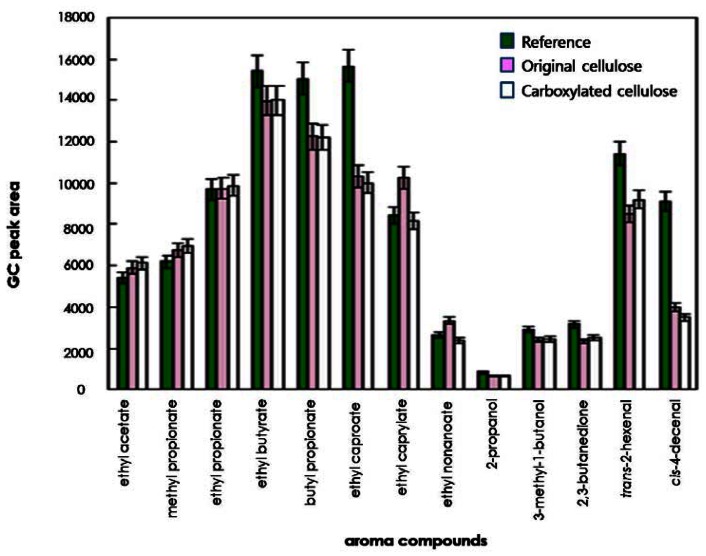
Comparison of release of odor compounds in hydrocolloid systems containing original or carboxylated cellulose and reference solution at pH 4.

**Figure 4.**
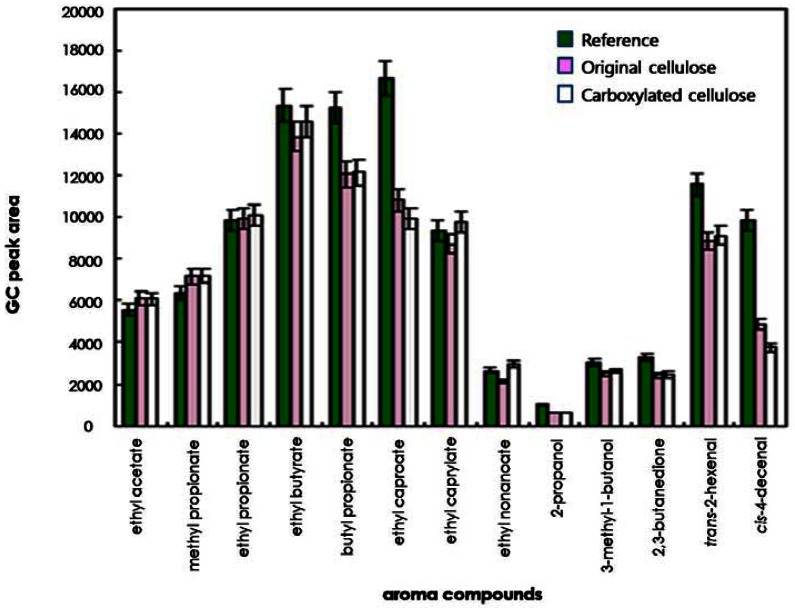
Comparison of release of odor compounds in hydrocolloid systems containing original or carboxylated cellulose and reference solution at pH 7.

**Figure 5.**
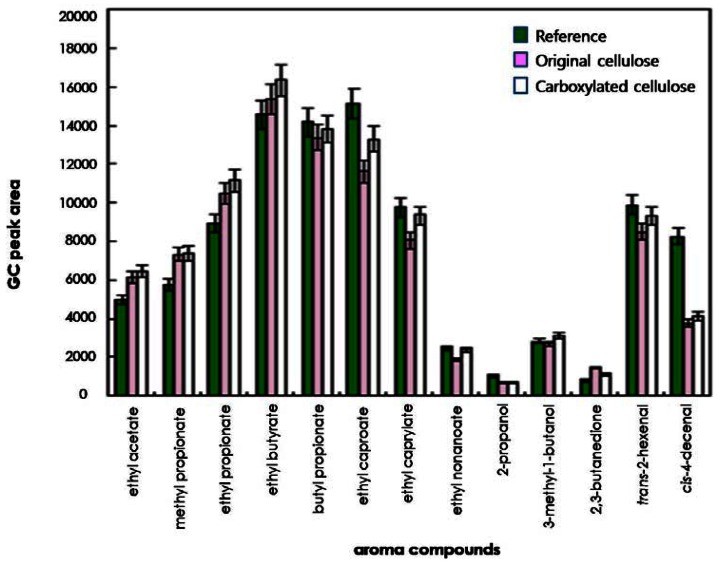
Comparison of release of odor compounds in hydrocolloid systems containing original or carboxylated cellulose and reference solution at pH 10.

To compare the effects of original and regioselectively carboxylated cellulose on the release of odorants, the quantities of 13 odorants released into the headspace were determined on the basis of their GC peak areas. As shown in Figures 3 and 4, for some compounds such as ethyl caproate, *trans*-2-hexenal, and *cis*-4-decenal, the GC peak areas of the reference solution (water system) were considerably larger than those of the original cellulose solution and the carboxylated cellulose solution adjusted to pH 4 and 7. However, for other compounds (ethyl acetate, methyl propionate, ethyl propionate, ethyl butyrate, and 2,3-butanedione), the GC peak areas of the cellulose-added hydrocolloid solutions (original and carboxylated cellulose solutions) adjusted to pH 10 were significantly larger than those of the reference solution (Figure 5). On the other hand, for butyl propionate, ethyl nonaoate, 3-methyl-1-butanol, and *trans*-2-hexenal, the GC peak areas between the cellulose-added hydrocolloid solution controlled to pH 10 and the reference solution were not significantly different (ANOVA and Duncan's test, P < 0.05). Furthermore, for ethyl caproate, ethyl caprylate, and ethyl nonanoate, the release of odorants in the original cellulose and the carboxylated cellulose solutions adjusted to pH 4, 7, and 10 were significantly different from each other (ANOVA and Duncan's test, P < 0.05). However, there was no considerable difference between original cellulose-containing and carboxylated-cellulose containing systems. Only minor difference could be found in the release of relatively long-chain esters such as ethyl caprylate and ethyl nonanoate.

In general, polysaccharides used could have an effect on the release of most odor compounds. Accordingly, the release of most odor compounds was larger in water (reference solution) than in the hydrocolloid solutions. In particular, 2-propanol and 3-methyl-1-butanol showed lower release in hydrocolloid model systems containing original and carboxylated cellulose in this study. Hydrogen bonding could have occurred between the hydroxyl group of 2-propanol or 3-methyl-1-butanol and the hydroxyl group and/or carbonyl group of cellulose, leading to increased retention of those compounds in the hydrocolloid model systems [3,21]. Also, the amounts of 2-propanol, 3-methyl-1-butanol, and 2,3-butanedione released into the headspace were less than those of any other odor compounds in hydrocolloid model systems. In the presence of polysaccharides, competition between the polar molecules and polysaccharides could occur to bind the water molecules, which could lead to an increased release of the odor compound to the gas phase [3,21]. Also, the release of the relatively nonpolar compounds (including ethyl butyrate, butyl propionate, ethyl caproate, *trans*-2-hexenal, and *cis*-4-decenal) was decreased by about 10%–40% in the hydrocolloid systems containing original and carboxylated cellulose. It was assumed that this phenomenon was due to the formation of hydrophobic agglomerates, that is, hydrophobic regions or nonpolar micelles by nonpolar odorants [3,22].

### 3.2. Influence of pH on the Odor Release in Hydrocolloid Systems

SHS-GC analysis was used to investigate the release of diverse odorants according to pH in hydrocolloid model systems (Tables 2 and 3). In the case of short and medium chain-esters (ethyl acetate, methyl propionate, ethyl propionate, ethyl butyrate, butyl propionate, ethyl caproate) and alcohols (2-propanol, 3-methyl-1-butanol), in particular, ethyl butyrate and 3-methyl-1-butanol, the release from the original and carboxylated cellulose solutions controlled to pH 10 was significantly higher than that from those adjusted to pH 4 and 7. Hansson *et al.*[23] investigated the effect of pH on the release of odor compounds from a soft drink model system. They found that changes to the pH values obtained by adding hydrochloric acid and citric acid had an effect on the release of esters. It was interesting to note that the release of 2,3-butanedione from both original and carboxylated cellulose solutions was increased at pH 4 and 7 compared to that at pH 10 by about 70% and 130%, respectively. The oxygen atoms at the carbonyl positions could carry more partially negative charges at higher pH, probably leading to stronger hydrogen bonds with hydroxyl groups of polysaccharides as well as those of water molecules.

## 4. Conclusions

The release of odor compounds in hydrocolloid systems at different pHs was determined using SHS-GC analysis. The release of most odor compounds was changed by addition of cellulose and carboxylated cellulose. This could be due to the chemical and physical interactions with original and carboxylated cellulose molecules affecting the release of odorants. However, there was no considerable difference in the release of most compounds, except for relatively long-chain esters such as ethyl caprylate and ethyl nonanoate, between original cellulose-containing and carboxylated-cellulose containing systems. On the other hand, the release of some odor compounds, such as ethyl propionate, ethyl butyrate, ethyl caproate ethyl nonanoate, and 2,3-butanedione, could be affected by pH and varied according to the odor compounds.

## Figures and Tables

**Table 1.** Physicochemical properties of odor compounds.

**Compounds**	**Density (a)**	**Boiling Point (a) (°C)**	**Molecular Weight (b)**	**Log *P*(c)**
2-Propanol	0.781(25 °C)	82.5	60.1	0.16 ± 0.19
2,3-Butanedione	0.990(15 °C)	88	86.09	–1.33 ± 0.33
Methyl propionate	0.915(20 °C)	79.7	88.11	0.71 ± 0.20
Ethyl acetate	0.902(20 °C)	77	88.11	0.71 ± 0.20
Ethyl propionate	0.891(20 °C)	99	102.13	1.24 ± 0.21
3-Methyl-1-butanol	0.819(19 °C)	113–114	88.15	1.22 ± 0.19
Ethyl butyrate	0.879(20 °C)	120–121	116.16	1.77 ± 0.21
*trans*-2-Hexenal	•	•	98.14	1.58 ± 0.28
Butyl propionate	0.875(20 °C)	146.8	130.18	2.30 ± 0.21
Ethyl caproate	0.873(20 °C)	166–167	144.21	2.83 ± 0.21
*cis*-4-Decenal	•	•	154.25	3.77 ± 0.24
Ethyl caprylate	0.878(17 °C)	207–209	172.26	3.90 ± 0.21
Ethyl nonanoate	•	•	188.29	4.43 ± 0.21

(a) The Merck Index-An encyclopedia of chemicals, drugs, and biologicals (13th edition), published by Merck Research Laboratories Division of MERCK & CO., Inc. (Whitehouse Station, NJ, USA);

(b) http://webbook.nist.gov/chemistry/name-ser.html;

(c) Advanced Chemistry Development, Inc. (http://www.acdlabs.com/ilab).

**Table 2.** Release of odor compounds in original cellulose hydrocolloid system adjusted to pH values of 4, 7, and 10.

**Odor Compounds**	**GC Peak Area**		

	**pH 4**	**pH 7**	**pH 10**
**Esters**			
Ethyl acetate	5,910 ± 580 a (A)	6,130 ± 190 a	6,140 ± 110 a
Methyl propionate	6,750 ± 370 a	7,160 ± 190 a	7330 ± 270 a
Ethyl propionate	9,740 ± 670 a	9,940 ± 360 a	10,480 ± 260 a
Ethyl butyrate	14,000 ± 600 b	13,900 ± 950 b	15,400 ± 190 a
Butyl propionate	12,200 ± 1,100 a	12,100 ± 1,400 a	13,400 ± 890 a
Ethyl caproate	10,300 ± 970 a	10,800 ± 1,300 a	11,600 ± 1,900 a
Ethyl caprylate	10,300 ± 2,500 a	8,700 ± 1,200 a	8,050 ± 400 a
Ethyl nonanoate	3,340 ± 1,000 a	2,140 ± 280 ab	1,890 ± 340 b
**Alcohols**			
2-Propanol	659 ±28 a	640 ± 16 a	677 ± 24 a
3-Methyl-1-butanol	2,410 ± 150 a	2,550 ± 61 a	2,700 ± 180 a
**Ketones**			
2,3-Butanedione	2,360 ± 240 a	2,460 ± 83 a	1,440 ± 28 b
**Aldehydes**			
*trans*-2-Hexenal	8,510 ± 820 a	8,870 ± 900 a	8,480 ± 41 a
*cis*-4-Decenal	3,970 ± 940 a	4,890 ± 940 a	3,770 ± 720 a

(A) The same letter (a/b) indicates that the mean values in a row are not significantly different at P < 0.05 between the different pH conditions.

**Table 3.** Release of odor compounds in carboxylated cellulose hydrocolloid system adjusted to pH values of 4, 7, and 10.

**Odor Compounds**	**GC Peak Area**		

	**pH 4**	**pH 7**	**pH 10**
**Esters**			
Ethyl acetate	6,120 ± 130 a (A)	6,100 ± 240 a	6,450 ± 620 a
Methyl propionate	6,960 ± 230 a	7,180 ± 160 a	7,370 ± 540 a
Ethyl propionate	9,860 ± 290 b	10,100 ± 310 b	11,100 ± 730 a
Ethyl butyrate	14,000 ± 340 b	14,600 ± 230 b	16,300 ± 830 a
Butyl propionate	12,200 ± 580 a	12,100 ± 180 a	13,800 ±1,800 a
Ethyl caproate	10,000 ± 520 b	9,940 ± 350 b	13,300 ± 1,500 a
Ethyl caprylate	8,180 ± 620 a	9,780 ±1300 a	9,350 ± 650 a
Ethyl nonanoate	2,370 ± 310 b	2,940 ± 200 a	2,410 ± 73 b
**Alcohols**			
2-Propanol	654 ± 57 a	642 ± 46 a	691 ± 150 a
3-Methyl-1-butanol	2,470 ± 110 b	2,640 ± 100 ab	3,110 ± 420 a
**Ketones**			
2,3-Butanedione	2,510 ± 92 a	2,470 ± 150 a	1,090 ± 140 b
**Aldehydes**			
*trans*-2-Hexenal	9,210 ±900 a	9,110 ± 860 a	9,310 ± 990 a
*cis*-4-Decenal	3,490 ± 890 a	3,790 ± 460 a	4,130 ± 480 a

(A) The same letter (a/b) indicates that the mean values in a row are not significantly different at P < 0.05 between the different pH conditions.
